# *Pneumocystis jirovecii* Pneumonia in Patients with Nephrotic Syndrome: Application of Lymphocyte Subset Analysis in Predicting Clinical Outcomes

**DOI:** 10.1155/2020/4631297

**Published:** 2020-02-20

**Authors:** Yang Liu, Ke Zheng, Yecheng Liu, Huadong Zhu

**Affiliations:** ^1^Department of Emergency Medicine, Peking Union Medical College Hospital, Peking Union Medical College, Chinese Academy of Medical Sciences, Beijing, China; ^2^Department of Nephrology, Peking Union Medical College Hospital, Peking Union Medical College, Chinese Academy of Medical Sciences, Beijing, China

## Abstract

**Purpose:**

With immunosuppressants being widely used, *Pneumocystis jirovecii* pneumonia (PCP) has been increasing and could be life-threatening among HIV-negative patients. This study aimed at identifying prognostic factors of PCP in patients with nephrotic syndrome.

**Methods:**

We retrospectively investigated patients with nephrotic syndrome who were diagnosed with PCP. The diagnosis of PCP was based on clinical manifestations, radiological findings, and microbiological confirmatory tests. Predictors of outcome were determined with multivariate logistic regression analysis.

**Results:**

A total of 57 patients were included in this study. The PCP mortality was 33.3%, which increased to 48.6% if ICU admission was required and to 60% when mechanical ventilation was needed. The T lymphocyte count and CD4/CD8 ratio independently predicted the outcome of PCP, so did the CD4^+^ T lymphocyte count (OR, 0.981; 95% CI, 0.967–0.996; *p*=0.001). The cut-off value of 71 cells/*μ*l for the CD4^+^ T lymphocyte count was determined to identify patients with poor prognosis. No association was found between PCP mortality and the type of immunosuppressant used.

**Conclusions:**

PCP is a fatal complication among nephrotic syndrome patients receiving immunosuppressive therapy. The CD4^+^ T lymphocyte count is suggested as an independent predictor of prognosis, which can be used clinically to identify patients with high risk of unfavorable outcomes.

## 1. Introduction

*Pneumocystis jirovecii* pneumonia (PCP), a pulmonary infection caused by *Pneumocystis jirovecii*, has been recognized as a kind of common opportunistic infection among immunocompromised patients. PCP has emerged as a concern since the epidemic of acquired immune deficiency syndrome (AIDS) in the 1980s and thus has been comprehensively studied in HIV-positive population [1]. However, since the early 2000s, more and more PCPs have been diagnosed among HIV-negative patients as the use of immunosuppressive agents increases in treating malignancies, autoimmune disorders, and inflammatory diseases, as well as in solid organ transplants [2–4]. In contrast to HIV-positive PCP, where the mortality has been reduced to less than 20%, PCP in HIV-negative patients has a poor prognosis with mortality rates of 30–55% as previously reported [5, 6]. Researches have been done in several HIV-negative cohorts to estimate the incidence and mortality rates of PCP in various underlying conditions [7–9]. While hematological malignancies and inflammatory diseases are considered as the main groups at risk of PCP, limited data are available in patients with nontransplant kidney diseases receiving immunosuppressants. Cases of PCP have been reported in patients with nephrotic syndrome [10, 11], but there are a few systemic investigations of PCP mortality among this group of people. Here, we aimed to assess the prognostic factors of PCP in patients with nephrotic syndrome by analyzing cases of PCP that occurred in this population from 2013 to 2018 in our institution with standardized methods of diagnosis and management.

## 2. Methods

### 2.1. Study Population and Definitions

We retrospectively investigated patients with nephrotic syndrome who were diagnosed with PCP and got treated at Peking Union College Medical Hospital, a tertiary care hospital in Beijing, China, between December 2013 and December 2018.

Nephrotic syndrome, which include a group of renal diseases with diverse etiologies, was specifically defined by the presence of heavy proteinuria (protein excretion greater than 3.5 g/24 hours), hypoalbuminemia (less than 30 g/L), and peripheral edema [12]. The diagnosis of PCP was based on clinical manifestations, such as fever, dyspnea or progressive hypoxemia, radiological findings, such as typical bilateral ground glass opacity on chest computed tomography, and microbiological confirmatory tests. A microbiological test was considered positive when *Pneumocystis jirovecii* cyst was detected in microscopic examination with Grocott's methenamine silver stain (GMS) or *Pneumocystis jirovecii* DNA (PCP-DNA) was revealed positive by polymerase chain reaction (PCR) assay of a sputum or bronchoalveolar lavage fluid (BALF) specimen. Patients with positive PCP-DNA specimens who did not have typical clinical and radiological features for PCP or who did not receive appropriate PCP treatments were excluded from our analysis.

### 2.2. Data Collection

Medical records were reviewed and collected from the hospital electronic database using the codes N04 for nephrotic syndrome and J17.3 for PCP, based on the International Classification of Diseases, 10^th^ Revision. Demographic features, clinical characteristics, comorbidities, immunosuppressive medications, renal pathology, laboratory values, treatments, and prognosis were recorded. Laboratory findings included complete blood counts, arterial blood gas analyses, blood biochemical indexes, T lymphocyte subsets, 1, 3-*β*-d-glucan levels, virus PCR assays, and procalcitonin (PCT). For laboratory values with more than one measurement, the worst values within 24 hours of hospital admission were acquired for analyses. Long-term corticosteroid therapy was defined as daily treatment for more than one month. The daily dosage of corticosteroids was expressed as the prednisone equivalent (1 mg of prednisone equals 0.8 mg of methylprednisolone which equals 1 mg of prednisolone). PCP mortality was defined as death rate caused by respiratory failure after PCP diagnosis.

### 2.3. Statistical Analysis

Statistical analyses were performed using Stata 14.0 SE or GraphPad Prism 6.0. The variables in the datasets were presented as mean ± SD or a number and proportion of the total (%). Continuous variables were analyzed by Mann-Whitney *U* test. Categorical variables were analyzed with Chi-square test or with Fisher's exact test, as appropriate. Logistic regression analysis was used to identify variables significantly associated with the outcome of PCP by estimating the odds ratios (OR) with 95% confidence intervals (CI). A two-tailed *p* value of <0.05 was considered significant.

## 3. Results

### 3.1. Patient Characteristics

Among 503 HIV-negative PCP cases, 57 individuals with nephrotic syndrome were included in our study (Table 1, [Supplementary-material supplementary-material-1]). The mean age of the patients was 48.1 ± 16.9 years old; 50.9% of cases were females. Hypertension, primary and secondary, existed in 30 (52.6%) patients. Only one (1.8%) patient had a history of chronic respiratory disease. They had suffered from nephrotic syndrome for a mean period of 23.7 ± 37.5 months at the onset of PCP. Most of them were receiving long-term corticosteroids (*n* = 56, 98.2%). Other immunosuppressive agents were used in combination with corticosteroids in 87.7% of patients, with cyclophosphamide (CTX, *n* = 31, 54.4%) and cyclosporine A (CsA, *n* = 13, 22.8%) being the most commonly used (Table 2). One (1.8%) patient had received trimethoprim/sulfamethoxazole (TMP-SMX) as prophylactic therapy for PCP. A total of 30 (52.6%) patients required mechanical ventilation, while 9 (15.8%) required continuous renal replacement therapy (CRRT) during hospitalization. Among all the included patients, 19 died from PCP, with a mortality rate of 33.3% ([Supplementary-material supplementary-material-1]).

### 3.2. Comparisons between Survivors and Nonsurvivors

In order to identify risk factors for PCP mortality, 38 survivors were compared with 19 nonsurvivors (Table 3). This showed no differences in age or in the duration of nephrotic syndrome between the two groups. Renal pathology was also not associated with prognosis. Three (7.9%) survivors were diagnosed with systemic lupus erythematosus (SLE) as the underlying disease for nephrotic syndrome, while 6 (31.6%) nonsurvivors suffered from SLE-related nephrotic syndrome (*p*=0.048). There was a trend that nonsurvivors had longer periods of corticosteroid administration compared with survivors, which was not statistically different. Initial body temperature and oxygen saturation (SpO_2_) were related to PCP mortality.

Lymphocyte subset analysis revealed that low cell counts of B lymphocyte (108.0 vs. 38.4, *p*=0.025), T lymphocyte (644.7 vs. 215.8, *p* < 0.001), and CD4^+^ T lymphocyte (305.4 vs. 65.2, *p* < 0.001) and a low CD4/CD8 ratio (1.04 vs. 0.48, *p* < 0.001) were associated with death from PCP among nephrotic syndrome patients. Blood urea was higher in nonsurvivors than survivors (*p*=0.045); however, serum creatinine level and estimated glomerular filtration rate (eGFR) did not differ between the two groups (Table 3). Combined virus infection, affecting both survivors and nonsurvivors, was detected in blood by PCR; CMV-DNA and EBV-DNA were shown positive in 50.0% and 22.2% of all cases, respectively. Bacterial coinfection also happened in both groups; *klebsiella pneumoniae*, the most common coinfecting bacterium, was found in 4 (7.0%) respiratory tract specimens of patients. Though the 1, 3-*β*-d-glucan assay is considered as a reliable indicator for PCP diagnosis [13], no significant difference was found in the level of 1, 3-*β*-d-glucan between survivors and nonsurvivors (Table 3).

All patients who had been diagnosed with PCP were treated immediately with TMP-SMX and dosed in the recommended range, above 15 mg/kg body weight (BW) per day. Compared with survivors, nonsurvivors had a higher rate of ICU admission (50.0% vs. 94.7%, *p*=0.001) and required more mechanical ventilation (31.6% vs. 94.7%, *p* < 0.001, Table 3). Extracorporeal membrane oxygenation (ECMO) support was used in one patient, who finally survived from PCP-related death.

### 3.3. Predictors of PCP Mortality

All covariates with a *p* value <0.05 (SLE, body temperature, SpO_2_, B lymphocyte count, T lymphocyte count, CD4^+^ T lymphocyte count, CD4/CD8 ratio, blood urea, ICU admission, and mechanical ventilation) were used for further analysis. By univariate regression, blood urea (*p*=0.061) and B lymphocyte count (*p*=0.145) were excluded from multivariate analysis. Mechanical ventilation and ICU admission were omitted because of their collinearity. CD4^+^ T lymphocyte count was correlated with T lymphocyte count (Pearson correlation coefficient = 0.97, *p* < 0.001) and CD4/CD8 ratio (Pearson correlation coefficient = 0.41, *p*=0.007), so they were analyzed separately. Multivariate regression analysis identified T lymphocyte count (OR, 0.992; 95% CI, 0.986–0.999) and CD4/CD8 ratio (OR, 0.023; 95% CI, 0.001–0.509) as independent predictors of PCP-related death (Table 4). Decreased CD4^+^ T lymphocyte count alone also predicted an unfavorable outcome (OR, 0.981; 95% CI, 0.967–0.996; *p*=0.001). T lymphocyte count + CD4/CD8 ratio model was not superior to CD4^+^ T lymphocyte model in predictive performance (*p*=0.325, [Supplementary-material supplementary-material-1]).

**Figure 1:**
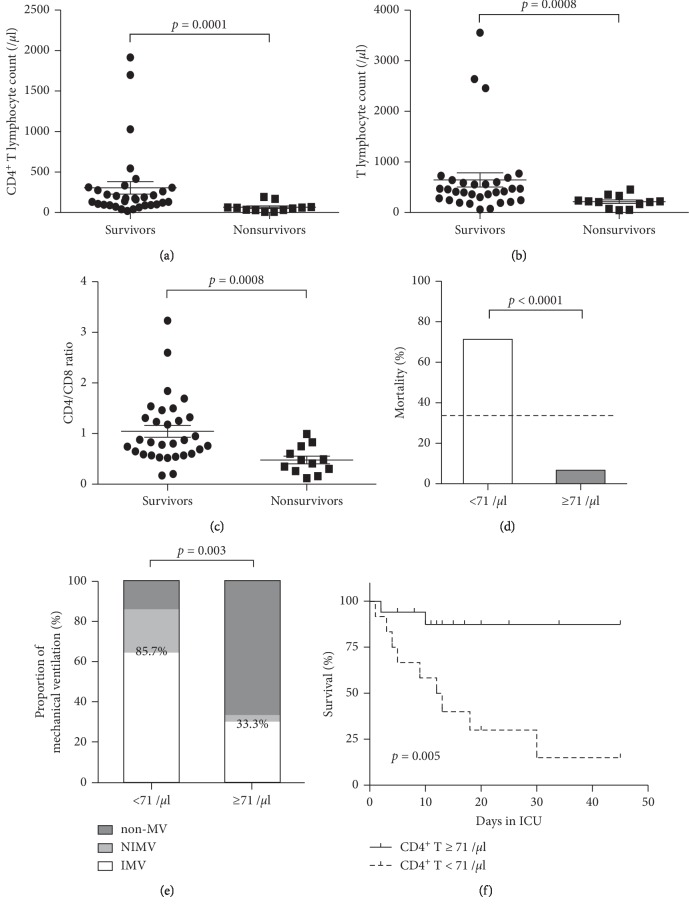
Predictors for PCP mortality and the influence on clinical course; (a–c) CD4^+^ T lymphocyte counts, T lymphocyte counts, and CD4/CD8 ratios in survivors and nonsurvivors. Error bars, mean ± SEM. (d) Mortality in patients with CD4^+^ T lymphocyte counts <71/*μ*l and ≥71/*μ*l. The dashed line indicates overall mortality of all patients (33.3%). (e) Proportion of mechanical ventilation in patients with CD4^+^ T lymphocyte counts <71/*μ*l and ≥71/*μ*l. Percentages (85.7% vs. 33.3%) shown in the graph represent the proportions of mechanical ventilation including both noninvasive and invasive ventilation. (f) Survival analysis of in-ICU patients stratified for CD4^+^ T lymphocyte counts <71/*μ*l and ≥71/*μ*l. *p* value, log-rank test. Non-MV: mechanical ventilation not applied, NIMV: noninvasive mechanical ventilation, and IMV: invasive mechanical ventilation.

The CD4^+^ T lymphocyte counts, as well as T lymphocyte counts and CD4/CD8 ratios, were significantly lower in nonsurvivors compared with survivors (Figures 1(a)–1(c)). The potential cut-off values for CD4^+^ T lymphocyte counts were computed ([Supplementary-material supplementary-material-1]), and 71/*μ*l (71 × 10^6^/L), with sensitivity and specificity both above 80%, was identified and suggested for predictive purpose. As is shown in Figure 1(d), the mortality rate was significantly higher among patients with CD4^+^ T lymphocyte counts lower than 71/*μ*l, compared to those with CD4^+^ T lymphocyte counts ≥ 71/*μ*l (71.4% vs. 6.67%, *p* < 0.0001). In addition, CD4^+^ T lymphocyte counts below 71/*μ*l, also indicated an increased need for respiratory support and a dramatic decrease in survival within ICU (Figure 1(e) and 1(f)). Among ventilated individuals, the rate of invasive mechanical ventilation (IMV) was relatively lower in patients with CD4^+^ T lymphocyte counts < 71/*μ*l when compared to those with CD4^+^ T lymphocyte counts ≥ 71/*μ*l (75% vs. 90%, Figure 1(e)), which may indicate the worry of ventilator-associated pneumonia for patients with severe lymphocytopenia.

## 4. Discussion

Infection, especially bacterial infection, has long been considered as one of the most common complications of nephrotic syndrome [14, 15]. However, few have studied opportunistic infection and no reliable data is available on *Pneumocystis jirovecii* infection among patients with nephrotic syndrome. Here, we investigated the clinical characteristics and outcomes of PCP in nephrotic syndrome patients over the past five years to identify prognostic factors of mortality in this specific population.

Nephrotic syndrome is mostly caused by primary glomerular diseases, while it may also present as one of the manifestations from systemic conditions [12, 16]. SLE is a common secondary cause of nephrotic syndrome. In our study, SLE was recognized as the underlying disease in 15.8% of all cases. Intriguingly, the mortality rate of PCP was doubled in SLE-nephrotic syndrome patients (66.7%) compared with overall mortality (33.3%). One possible explanation might be that SLE patients were administrated with corticosteroids for longer periods (SLE vs. non-SLE, 41.6 ± 27.1 vs. 7.2 ± 9.1 months; [Supplementary-material supplementary-material-1]) and may experience more severe immune suppression, which merit further investigation.

Patients with nephrotic syndrome are more susceptible to infection because of reduced serum immunoglobulin G and complement levels, suppressed T cell immunity, and administration of immunosuppressive therapy [17–19]. Previous studies have suggested the use of corticosteroids is associated with the development of PCP. Even daily doses of prednisone between 16 and 20 mg for at least four weeks were believed to increase the risk of PCP in HIV-negative patients [20]. An average daily dose of 47.3 ± 32.8 mg equivalent prednisone was reported at the onset of PCP among HIV-negative patients [21]. In nephrotic syndrome cohort who developed PCP, we showed that nearly all patients (98.2%) were receiving long-term corticosteroids. Prior to the diagnosis of PCP, 91.2% were dosed with 20 mg/d of equivalent prednisone or more, and the mean duration of corticosteroid administration was 12.6 ± 18.1 months. In addition to corticosteroids, other immunosuppressive agents, such as CTX, CsA, mycophenolate mofetil (MMF), and methotrexate (MTX), also have been reported to be associated with PCP development [22, 23]. A retrospective study found that three out of 32 IgA nephropathy patients developed severe pneumonia with *Pneumocystis jirovecii* infection when administrated with MMF, which did not occur in CTX-treated patients [24]. However, in our study with nephrotic syndrome patients, PCP was developed regardless of the type of immunosuppressant used. CTX was the most commonly used agent in our cohort who had developed PCP. No association was found between PCP-related death and the type of immunosuppressant. Besides, lack of specific guidelines for chemoprophylaxis also contributes to increased occurrence of PCP [23]. Stern et al. [25] suggested that prophylaxis for PCP using TMP-SMX was highly effective among HIV-negative immunocompromised patients and thus should be considered in this population, especially those with leukemia and transplants. Nevertheless, there are no current guidelines for PCP prophylaxis in nephrotic syndrome adults. Among nephrotic syndrome patients who developed PCP, we found that 98.2% (56/57) of individuals had not been prescribed PCP prophylaxis; the only one patient (1/57), receiving prophylaxis before the onset of PCP, survived from PCP-related death. Therefore, the present study is providing indirect evidence that PCP prophylaxis is important in immunocompromised hosts, including nephrotic syndrome patients.

It is well established that HIV-positive patients with immune deficiency are at a high risk of PCP and lymphocytopenia. Decreased CD4^+^ T lymphocytes, especially, is believed to be associated with the occurrence and outcome of PCP [26, 27]. However, the relationship between PCP and low CD4^+^ T lymphocyte count is less obvious in HIV-negative patients [9, 21], perhaps because of the heterogeneity of underlying diseases. In our study, low CD4^+^ T lymphocyte count was identified as a significant predictor of PCP mortality among nephrotic syndrome patients. The cut-off value for CD4^+^ T lymphocyte count (71/*μ*l, or 71 × 10^6^/L) was defined with a satisfactory specificity and sensitivity. This could be quite helpful in clinical practice for physicians to identify patients with high risk of unfavorable outcomes. In fact, the combination of T lymphocyte count and CD4/CD8 ratio was a better predictive model compared with CD4^+^ T lymphocyte alone, but the difference was slight, and single parameter could be more convenient for clinical use. In addition, mortality might also be associated with a reduction in B lymphocyte count; however, this observation was not confirmed in multivariate analysis. It is possible that the impact of B lymphocytes on prognosis may be insignificant under the condition of T lymphocytopenia, whereas B lymphocytes may show importance with a normal number of T lymphocytes, as is suggested in several studies [28–30].

The level of blood urea usually indicates protein catabolism and a negative nitrogen balance. It is an important parameter for assessing the prognosis of community-acquired pneumonia in CURB-65, a pneumonia severity scoring system [31]. It has also been reported that blood urea level is associated with mortality in HIV-negative PCP [32]. Among nephrotic syndrome patients, our data showed that the blood urea level was higher in nonsurvivors, which may reflect a worsened metabolic and nutritional status, given that the renal function (serum creatinine and eGFR) was comparable between survivors and nonsurvivors. ICU admission and mechanical ventilation are frequently reported to be associated with a poor prognosis of PCP [5, 33]. In HIV-negative patients, ICU admission rate was reported to be 41.7% and 60% in two studies [33, 34]; the in-ICU mortality of 53% was reported in another recent study [7]. With regard to mechanical ventilation, it occurred to 40–54% of HIV-negative patients [34, 35], and the mortality rate increased to 69.3% compared with the overall mortality of 35.8% in one report [32]. In comparison, among nephrotic syndrome patients who developed PCP, the rates of ICU admission and in-ICU mortality were 64.9% and 48.6%, respectively. Mechanical ventilation was applied to 52.6% of patients, and 60% of ventilated patients died from PCP. Our findings could provide a general perception of intensive care management and outcome of PCP in this specific subgroup of HIV-negative population.

Our study has several limitations. It was a retrospective study and data were collected from a single institution. As medical records were reviewed, some incomplete records with missing values may affect the results of the study. Besides, for some nephrotic syndrome patients included, the underlying disorders had not been identified; thus, we were unable to figure out the possible association between different etiologies and PCP development. Finally, in some patients, *Pneumocystis jirovecii* was only detected by PCR without the confirmation by GMS; a false positive result might be an issue. However, the diagnosis of PCP was made based on typical clinical manifestations and radiological findings in addition to microbiological tests, which maximally reduced the chances of false positive results.

## 5. Conclusions

In summary, PCP is a fatal complication in nephrotic syndrome patients receiving immunosuppressive therapy. Lymphocyte subset analysis may serve as a stratifying tool to identify those patients at high risk of PCP-related death. Decreased CD4^+^ T lymphocyte count could be a useful predictor of PCP mortality among nephrotic syndrome patients, which can be easily used clinically. More robust evidence from well-designed prospective studies is needed to validate the current findings.

## Figures and Tables

**Table 1:** Patient characteristics and managements.

Variables	*N* = 57
Age (years)	48.1 ± 16.9
Sex (female)	29 (50.9%)
Duration of nephrotic syndrome (months)	23.7 ± 37.5
Ever-smokers	10 (17.5%)
Comorbidity	
Chronic respiratory disease	1 (1.8%)
Hypertension	30 (52.6%)
Diabetes mellitus	13 (22.8%)
Type 2 diabetes	4 (7.0%)
Steroid-induced diabetes	9 (15.8%)
Heart disease	7 (12.3%)
Coronary heart disease	4 (7.0%)
Arrhythmia	1 (1.8%)
Hypertensive heart disease	1 (1.8%)
Hypothyroid heart disease	1 (1.8%)
Use of immunosuppressants	
Long-term corticosteroids	56 (98.2%)
Duration of corticotherapy (months)	12.6 ± 18.1
Other immunosuppressive agents	50 (87.7%)
Diagnosis of PCP	
PCR	56 (98.2%)
GMS	8 (14.0%)
Managements	
ICU admission	37 (64.9%)
Mechanical ventilation	30 (52.6%)
CRRT	9 (15.8%)
ECMO	1 (1.8%)

PCP: *Pneumocystis jirovecii* pneumonia, PCR: polymerase chain reaction, GMS: Grocott's methenamine silver stain, ICU: intensive care unit, CRRT: continuous renal replacement therapy, and ECMO: extracorporeal membrane oxygenation. Data were presented as mean ± SD or numbers (%).

**Table 2:** Exposure to immunosuppressants before diagnosis of PCP.

Immunosuppressants	*N* = 50
CTX	24 (48%)
CsA	10 (20%)
MMF	4 (8%)
FK506	2 (4%)
MTX	1 (2%)
TwHF	1 (2%)
LEF	1 (2%)
CTX + CsA	3 (6%)
CTX + TwHF	2 (4%)
CTX + MMF	1 (2%)
CTX + HCQ	1 (2%)

CTX: cyclophosphamide, CsA: cyclosporine A, MMF: mycophenolate mofetil, FK506: tacrolimus, MTX: methotrexate, TwHF: tripterygium wilfordii hook F, LEF: leflunomide, and HCQ: hydroxychloroquine. Data were presented as numbers (%).

**Table 3:** Comparisons of clinical features, laboratory parameters, and managements between survivors and nonsurvivors.

Variables	Survivors (*N* = 38)	Nonsurvivors (*N* = 19)	*p* value
Age (years)	47.6 ± 17.1	48.9 ± 17.3	0.859
Sex (female)	18 (47.4%)	11 (57.9%)	0.454
Duration of nephrotic syndrome (months)	20.2 ± 37.1	30.8 ± 38.2	0.202
Ever-smokers	7 (18.4%)	3 (15.8%)	1.000
Hypertension	20 (52.6%)	10 (52.6%)	1.000
Diabetes mellitus	10 (26.3%)	3 (15.8%)	0.510
SLE as the underlying disease	3 (7.9%)	6 (31.6%)	0.048
*Renal pathology*			0.180
Membranous nephropathy	18 (47.4%)	8 (42.1%)
Mesangial proliferative glomerulonephritis	5 (13.2%)	0 (0.0%)
Minimal change nephropathy	3 (7.9%)	0 (0.0%)
Focal segmental glomerulosclerosis	2 (5.3%)	2 (10.5%)
Others	10 (26.3%)	9 (47.4%)
*Use of immunosuppressants*			
Duration of corticosteroid administration (months)	8.9 ± 13.6	19.9 ± 24.0	0.079
Dose of corticosteroids before symptom onset			
Low doses (<20 mg/d)	2 (5.3%)	3 (15.8%)	0.321
High doses (≥20 mg/d)	36 (94.7%)	16 (84.2%)	0.321
Immunosuppressive agents	33 (86.8%)	17 (89.5%)	1.000
CTX	21/33 (63.6%)	10/17 (58.5%)	0.767
CsA	10/33 (30.3%)	3/17 (17.6%)	0.499
*Symptoms*			
Fever	35 (92.1%)	18 (94.7%)	1.000
Cough	33 (86.8%)	18 (94.7%)	0.652
Dyspnea	32 (84.2%)	19 (100%)	0.164
*Initial vital signs*			
Body temperature ( C)	37.09 ± 0.87	37.87 ± 0.82	0.002
Heart rate (beats per min)	94.7 ± 17.0	99.3 ± 20.7	0.436
Respiratory rate (breaths per min)	24.8 ± 8.1	27.1 ± 7.8	0.189
Mean arterial pressure (mmHg)	90.5 ± 11.1	86.2 ± 16.4	0.088
SpO_2_ (%)	93.5 ± 4.9	89.8 ± 7.0	0.048
*Laboratory findings*			
pH	7.44 ± 0.06	7.44 ± 0.06	0.961
PaO_2_ (mmHg)	63.6 ± 21.2	54.4 ± 9.7	0.187
PaCO_2_ (mmHg)	35.3 ± 5.6	33.8 ± 5.4	0.259
pO_2_ (A-a) (mmHg)	45.4 ± 20.1	58.0 ± 8.7	0.096
Blood leukocyte count (10^9^/L)	10.00 ± 4.55	9.48 ± 5.49	0.636
Blood neutrophil count (10^9^/L)	8.85 ± 4.23	8.74 ± 5.32	0.846
Blood lymphocyte count (10^9^/L)	1.12 ± 1.91	0.52 ± 0.38	0.095
B Lymphocyte count (*μ*l)	108.0 ± 130.0	38.4 ± 51.4	0.025
T Lymphocyte count (*μ*l)	644.7 ± 781.7	215.8 ± 123.0	<0.001
CD4^+^ T lymphocyte count (*μ*l)	305.4 ± 437.8	65.2 ± 57.6	<0.001
CD8^+^ T lymphocyte count (*μ*l)	302.0 ± 341.6	139.8 ± 79.6	0.074
CD4/CD8 ratio	1.04 ± 0.66	0.48 ± 0.27	<0.001
Albumin (g/L)	23.6 ± 5.0	22.1 ± 4.2	0.109
Urea (mmol/L)	10.18 ± 6.36	14.28 ± 8.79	0.045
Creatinine (*μ*mol/L)	142.7 ± 129.9	167.7 ± 121.4	0.108
eGFR (mL/min·1.73 m2)	82.5 ± 67.1	54.9 ± 36.2	0.098
Hypersensitive C-reactive protein (mg/L)	75.19 ± 78.98	122.80 ± 119.6	0.156
Lactate dehydrogenase (U/L)	559.0 ± 194.3	807.5 ± 416.4	0.091
1,3-*β*-d-Glucan assay (pg/ml)	1383 ± 1043	1045 ± 911	0.224
Positive blood CMV-DNA by PCR	17/38 (44.7%)	11/18 (61.1%)	0.391
Positive blood EBV-DNA by PCR	9/37 (24.3%)	3/17 (17.6%)	0.732
PCT ≥ 0.5 ng/ml	14 (36.8%)	12 (63.2%)	0.091
*Managements*			
ICU admission	19 (50.0%)	18 (94.7%)	0.001
Mechanical ventilation	12 (31.6%)	18 (94.7%)	<0.001
IMV	10 (26.3%)	15 (78.9%)	
NIMV	2 (5.3%)	3 (15.8%)	
CRRT	4 (10.5%)	5 (26.3%)	0.143

SLE: systemic lupus erythematosus, CTX: cyclophosphamide, CsA: cyclosporin A, SpO_2_: oxygen saturation, CMV: cytomegalovirus, EBV: Epstein-Barr virus, PCT: procalcitonin, IMV: invasive mechanical ventilation, and NIMV: noninvasive mechanical ventilation. Data were presented as mean ± SD or numbers (%).

**Table 4:** Clinical parameters associated with mortality from PCP.

Variables	Odds ratio	95% CI	*p* value
T Lymphocyte count	0.992	0.986–0.999	0.021
CD4/CD8 ratio	0.023	0.001–0.509	0.017

## Data Availability

The datasets analyzed during the current study are available from the corresponding author on reasonable request.

## Supplementary Materials

Supplementary Materials Table S1: underlying diseases in cases of PCP among HIV-negative patients. Table S2: overall mortality and mortality in subgroups of the studied population. Table S3: performance of models regarding the prediction of PCP mortality. Table S4: potential alternative cut-off values of CD4^+^ T lymphocyte count and corresponding statistics related to the prediction of mortality in nephrotic syndrome patients with PCP. Figure S1: duration of corticosteroid administration before PCP diagnosis in SLE and non-SLE patients.
