# Supplementary material for: Pneumocystis jirovecii Pneumonia in Patients with Nephrotic Syndrome: Application of Lymphocyte Subset Analysis in Predicting Clinical Outcomes
Source: Can J Infect Dis Med Microbiol. 2020 Feb 20;2020:4631297. doi: 10.1155/2020/4631297 (PMC7054770; doi:10.1155/2020/4631297)
Supplement: Supplementary Materials — Table S1: underlying diseases in cases of PCP among HIV-negative patients. Table S2: overall mortality and mortality in subgroups of the studied population. Table S3: performance of models regarding the prediction of PCP mortality. Table S4: potential alternative cut-off values of CD4+ T lymphocyte count and corresponding statistics related to the prediction of mortality in nephrotic syndrome patients with PCP. Figure S1: duration of corticosteroid administration before PCP diagnosis in SLE and non-SLE patients. [file 4631297.f1.pdf]

Supplementary material

Table S1 Underlying diseases in cases of PCP among HIV-negative patients

Diseases	N = 503
Nephrotic syndrome	57 (11.3%)
Malignancies	205 (40.8%)
Autoimmune / Inflammatory diseases [a]	216 (42.9%)
Post-transplantation	13 (2.6%)
Others [a]	12 (2.4%)

[a] Nephrotic syndrome may be secondary to systemic inflammatory disorders and others. Only those cases without nephrotic syndrome development were counted.

Table S2 Overall mortality and mortality in subgroups of the studied population

	Mortality
Death	19/57 (33.3%)
ICU admission	18/37 (48.6%)
Mechanical ventilation	18/30 (60.0%)

Data were presented as fractions and percentages.

Table S3 Performance of models regarding the prediction of PCP mortality

	AUC	Standard Error	95% CI
$CD4^+$ T lymphocyte count	0.871	0.064	0.745-0.997
T lymphocyte count + CD4/CD8 ratio	0.889	0.065	0.763-1.000
T lymphocyte count	0.820	0.065	0.693-0.947
CD4/CD8 ratio	0.820	0.072	0.678-0.962

Abbreviations: AUC area under the curve (receiver operating characteristic).

Table S4 Potential alternative cut-off values of $CD4^+$ T lymphocyte count and corresponding statistics related to the prediction of mortality in nephrotic syndrome patients with PCP

Cutpoint	Sensitivity	Specificity	LR+	LR-
≥ 40	96.77%	41.67%	1.659	0.077
≥ 63	93.55%	66.67%	2.806	0.097
≥ 71	90.32%	83.33%	5.419	0.116
≥ 174	51.61%	91.67%	6.194	0.528

Abbreviations: LR+ positive likelihood ratio, *LR-* negative likelihood ratio.

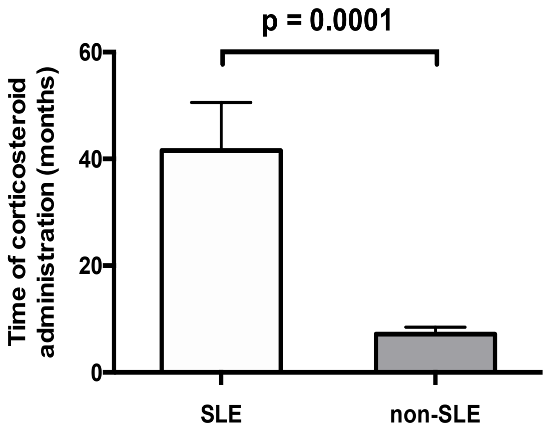

Fig. S1 Duration of corticosteroid administration before PCP diagnosis in SLE and non-SLE patients (Error bars, mean ± SEM)
